# The role of neuroticism and conscientious facets in academic motivation

**DOI:** 10.1002/brb3.2673

**Published:** 2022-07-14

**Authors:** Nikolas Apostolov, Madelyn Geldenhuys

**Affiliations:** ^1^ Australian College of Applied Psychology Sydney Australia; ^2^ University of Notre Dame Australia Fremantle Australia; ^3^ University of Johannesburg Johannesburg South Africa

**Keywords:** academic motivation, Big‐5, conscientiousness, facets, neuroticism, C. F. Halverson, Jr., G. A. Kohnstamm, & R. P. Martin (Eds.), *The developing structure of temperament and personality from infancy to adulthood* (pp. 139–150). Lawrence Erlbaum Associates, Inc

## Abstract

**Introduction:**

Personality differences have been demonstrated to influence an individual's academic performance in different ways. Notably, conscientiousness is the most consistent significant predictor of academic performance, while neuroticism shows inconsistent results.

**Objectives:**

This study aimed to determine the relationship between the facets of conscientiousness and neuroticism on academic motivation.

**Method:**

The study was conducted in Australia and consisted of 285 undergraduate students. The International Personality Item Pool and Motivated Strategies Learning Questionnaire were used to measure personality and motivation, respectively. Structural equation modeling results revealed that conscientiousness had the most significant relationship with academic motivation, while neuroticism was negatively related. The conscientious facets of self‐efficacy and achievement striving were positively related to academic motivation. The results also revealed that the anxiety facet of neuroticism was the only significant positive predictor for academic motivation, while depression and vulnerability were negatively related.

**Conclusion:**

This study reveals how personality facets contribute to academic motivation over assessing grades and superordinate factors alone. Trait‐level anxiety significantly contributes to academic motivation, helping us shed light on underlying mechanisms such as defensive pessimism, resulting in higher motivation due to fearing the worst.

## Graphical abstract

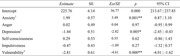

## 1 THE RELATIONSHIP OF NEUROTICISM, CONSCIENTIOUSNESS AND ACADEMIC MOTIVATION

Over the years, research has found that personality is equally important as intelligence for achieving high academic performance (Vedel, 2014). Understanding how personality affects academic motivation and performance is particularly important during the COVID‐19, BC, C1 pandemic, as most research has only studied how personality affects face‐to‐face learning (Chiu et al., 2021). Although personality is difficult to define, it may be described as the individually distinct qualities and characteristics of a person. Costa and McCrae (1992) identified five broad traits, the Big‐5, encompassing the human personality spectrum to make a more profound sense of personality. The five personality traits of conscientiousness, openness to experience, extraversion, agreeableness, and neuroticism (see Costa & McCrae, 1992) have emerged as the most used categorization of personality applied in many different contexts, including education, health, and workplaces. The Big‐5 has been demonstrated to not only be biological (Yamagata et al., 2006) but also predict job preference (Salgado, 1998), job performance (Peral & Geldenhuys, 2020), and academic performance (Trapmann et al., 2007) and is a predisposition to overall health and well‐being (Friedman & Kern, 2014). In their study, Trapmann et al. (2007) revealed that conscientiousness is the most reliable predictor of academic performance, whereas agreeableness, extraversion, and openness to experience were not significant predictors.

Trapmann et al. (2007) further suggested that research into neuroticism is essential because it does not clearly correlate with academic performance. Vedal (2014) argued that the six facets of neuroticism might have unique correlations with academic performance due to the individual facets relating in unique ways. One of the critical research outcomes is personality factors, for example, conscientiousness and neuroticism relating to grades. Studies show that personality predicts varying grades (see Komarraju & Karau, 2005; Vedal, 2014). Academic motivation over academic grade performance is useful in understanding performance and intent to perform. Research by Steinmayr et al. (2019) revealed that the different dimensions of academic motivation accounted for more variance in academic grades than prior achievement and intelligence. Although motivation is often not examined, Steinmayr et al. (2019) highlight how a student's degree of motivation influences their academic performance and predicts their grades. Therefore, this research contributes to the literature by investigating the facets of conscientiousness and neuroticism and their influence on academic motivation. This helps shed light on the trait‐level facets that enable motivation in academic performance.

### 1.1 Personality and academic performance

One of the most popular and scientifically grounded frameworks for personality is Big‐5 (Chamorro‐Premuzic & Furnham, 2003). Costa and McCrae et al. (1992) identified five main clusters in their data and mapped personality along a continuum of five basic dimensions. Expanding on the Big‐5 model, McCrae et al. (2010) created the Neuroticism, Extraversion, Openness, Conscientiousness, Agreeableness, Personality Inventory, which added six facets to each of the five traits. Neuroticism describes someone as anxious and impulsive, whereas extraversion is defined as being socially motivated and enthusiastic (McCrae & Costa, 1994). Openness to experience characterizes someone with an active imagination and is open to new ideas (Barford & Smillie, 2016). Agreeableness is defined as preferring cooperation over competitiveness, and conscientiousness typifies someone who is hard working and disciplined (Bamford & Davidson, 2017).

Chamorro‐Premuzic and Furnham (2003) revealed that conscientiousness was the strongest predictor of academic performance measured by grade point average (GPA) scores, whereas openness, extraversion, and agreeableness produced no significant correlations. Although neuroticism revealed a negative correlation with GPA scores, it produced no significant correlation with coursework (Chamorro‐Premuzic & Furnham, 2003). Furthermore, Hakimi et al. (2011) confirmed the findings from Chamorro‐Premuzic and Furnham (2003) by revealing that conscientiousness accounted for most of the variance in GPA scores. One study that examined the trait facets by Noftle and Robins (2007) confirmed the Chamorro‐Premuzic and Furnham (2003) and Hakimi et al. (2011) findings by revealing that conscientiousness was the only significant positive predictor for GPA scores in both high school and college. Noftle and Robins (2007) found that the conscientious facets of achievement striving, competence, and self‐discipline were the most significant predictors for GPA scores. Trapmann et al. (2007) revealed that conscientiousness produced a significant moderate positive correlation with GPA scores, which complements the findings by Chamorro‐Premuzic and Furnham (2003), Hakimi et al. (2011), and Noftle and Robins (2007). On the other hand, Trapmann et al. (2007) demonstrated that neuroticism was not a reliable predictor of academic performance, despite the Chamorro‐Premuzic and Furnham (2003) findings. Trapmann et al. (2007) argued that the neuroticism facets need to be further examined because they may produce different correlations.

### 1.2 Facets of neuroticism and academic motivation

Vedal (2014) argues that the lack of association in their meta‐analysis between neuroticism and academic performance is because some neuroticism facets might be beneficial. Exploring the facets of neuroticism may also explain the mixed findings by Chamorro‐Premuzic and Furnham (2003), who found that the neuroticism facets of anger and impulsiveness negatively correlated with exam scores. Furthermore, the impulsivity facet has a negative correlation with academic achievement as demonstrated by Herman et al. (2018). Echoing the need to explore the facets of neuroticism, Vedal (2014) believes that most of the literature has overlooked how the facets correlate with academic performance despite the probability that the facets are more robust predictors. As O'Súilleabháin et al. (2019) argue, although the six facets combine to create the higher‐order trait of neuroticism, each facet provides very different degrees of emotional instability.

In addition to mostly ignoring the facets, most of the literature only focuses on academic performance, not considering motivational factors like attitude toward study or test strategies (Chamorro‐Premuzic & Furnham, 2003; Komarraju & Karau, 2005). Academic motivation encapsulates the study habits, strategies, and perceived intrinsic value of the academic study. Komarraju and Karau (2005) revealed that although conscientiousness provided the strongest correlation with achievement, neuroticism was surprisingly the second‐highest predictor. Komarraju and Karau (2005) speculated that the anxiety facet might be motivating individuals to engage in compulsive preparation for the exam. Perhaps the participants who scored high in neuroticism in the Komarraju and Karau (2005) study engaged in defensive pessimism due to anxiety. Norem and Chang (2002) found that defensive pessimism predicted higher academic achievement than individuals who did not prepare for the worst.

Furthermore, a study by Turiano et al. (2013) revealed that the anxiety facet of neuroticism might act as a motivating factor for conscientious individuals to engage in regular health check‐ups and healthy living. The anxiety facet serving as a motivating factor argument falls in line with the defensive pessimism findings from Norem and Chang (2002) and the compulsive preparation theory in the Komarraju and Karau (2005) study. Therefore, neuroticism's anxiety facet may be acting as a motivating factor, whereas the impulsivity and vulnerability facets demonstrated a negative correlation.

In summation, previous research has demonstrated a positive link between conscientiousness and academic performance, whereas the role of neuroticism facets and how personality affects motivation to study is unclear. The few research papers that examined the facets of neuroticism found that impulsivity and vulnerability are negatively correlated with academic performance; however, anxiety may have a beneficial role due to anxiety‐induced compulsive preparation. Furthermore, most of the cited research is a decade old and did not examine students studying online. Understanding what personality traits predict academic motivation is fundamental because the academic landscape is changing as highlighted by Hall and Batty (2020), which states that students find it challenging to stay motivated while studying from home during COVID‐19. Therefore, this paper aims to examine the facets of neuroticism and how neuroticism correlates with academic motivation moderated by conscientiousness.

### 1.3 Objective and hypothesis

This paper's main objective is to investigate the relationships between conscientiousness, neuroticism, and academic motivation. This study also aims to investigate how the facets of conscientiousness and neuroticism relate to motivation to perform. The hypotheses for this paper are as follows.

**H1**: *The higher‐order trait of conscientiousness and its facets of (a) achievement striving, (b) competence, (c) dutifulness, (d) self‐efficacy, (e), deliberation, and (f) orderliness are positively related to academic motivation*.
**H2**: *The higher‐order trait of neuroticism and its facets of (a) depression, (b) impulsiveness, (c) anger, (d) vulnerability, and (e) self‐consciousness will negatively relate to academic motivation, whereas (f) anxiety will have a positive correlation*.

## 2 METHOD

### 2.1 Participants

Participants were psychology students from a higher education institution *N* = 285.^1^ Participation was voluntary and anonymous. The preanalysis screening revealed that after removing participants with missing data (1.68 %; *n* = 5) and participants who did not provide consent (3%, *n* = 9), the remaining 91% of the data were considered useable. Most of the participants were female (219), comprising 78.2% of the sample, 53 (18.9%) identified as male, four (1.4%) identified as nonbinary, and one (.4%) preferred not to say. Most participants were between the 18 and 25 age range (125; 44.6%), 61 were between the age range of 26 and 35 (21.8%), 56 were between 36 and 45 years (20%), 46 were between 46 and 55 years (27%), and only seven (2.5%) were over 55 years of age. The participants in this study came from diverse backgrounds, including African (2.5%), Samoan (0.8%), and Asian (3.5%). Most of the students were studying online (163; 58.2%), only 29 (10.4%) were studying on campus, and 85 (30.4%) were studying blended. Finally, most participants studied full‐time (180), comprising 64.3% of the sample, and 97 (34.6%) were studying part‐time. Sixty‐one (21.8%) of the students in this sample worked full‐time, while 80 (28.6%) worked part‐time, 71 (25.4%) of the sample had casual employment, and 64 (22.9%) were unemployed.

### 2.2 Measures

#### 2.2.1 Personality

The International Personality Item Pool (IPIP) is a 120‐item questionnaire designed to measure five traits: openness to experience, conscientiousness, extraversion, agreeableness, and neuroticism. The IPIP also measures each of the five traits’ six facets using a 5‐point Likert‐type scale (1 = *not at all like me* and 5 = *very much like me*), with high scores indicating higher levels of the facet assessed. Sample items include completing tasks successfully and fear for the worst (Maples et al., 2014). The Cronbach's alpha score for the IPIP in this study is *α* = .80, which demonstrates excellent reliability according to the reliability range by Ursachi et al. (2015). For each of the traits measured in this study, the reliability scores were neuroticism *α* = .90, extraversion *α* = .92, openness to experience *α* = .82, agreeableness *α* = .83, and conscientiousness *α* = .90.

#### 2.2.2 Academic motivation

Despite psychometric issues around some of its subscales, the full 81‐item Motivated Strategies For Learning Questionnaire (MSLQ) has demonstrated good validity and reliability (Crede & Phillips, 2011). Removing the problematic subscales, Pintrich and De Groot (1990) created a 44‐item questionnaire that uses a 7‐point Likert‐type scale (1 = *not at all true of me* and 7 = *very true of me*), with high scores indicating high levels of academic motivation. The MSLQ measures academic motivation across five subscales: self‐efficacy (e.g., “I think I will receive a good grade in this class”), intrinsic value, test anxiety, cognitive strategy use, and self‐regulation. The subscales are summed to create two dimensions, self‐regulation and motivation. In the present study, the Cronbach's alpha results for the MSLQ revealed that it has very good internal consistency *α* = .91.

#### 2.2.3 Procedure

Following the approval from a Human Resource Ethics Committee (approval number: 711041220), we sent out internal invitations to first‐year undergraduate students studying at an Australian Higher Education Institution. The participants were invited to use the Institution's system to complete the survey hosted via Qualtrics. After the participants read the information statement, they were asked to provide consent. Participants were informed that they could withdraw at any time by closing their browser window and that there was a services sheet if they experienced any mental distress. After the participants provided initial consent, they were asked to provide demographic information and complete the IPIP and MSLQ surveys. They were debriefed and asked to reconfirm their consent to analyze the data.

#### 2.2.4 Statistical analysis

The statistical analysis was conducted through Statistical Package for Social Sciences statistical program 25 and the RStudio (R Core Team, 2016) using the psych (Revelle, 2019), lavaan (Rosseel, 2012), and structural equation modeling (SEM; Fox et al., 2012) packages. Before any analysis was conducted, the data were screened for outliers using the standard deviation of (< 3 >) and problems with normality assumptions. Little's missing completely at random (MCAR) test was calculated for missing data. Descriptive statistics were calculated to reveal the mean, median, standard deviation, skewness, and kurtosis. The Shapiro–Wilk significance test of *p* > .05, as suggested by Hinton et al. (2014), was used to determine if the data were normally distributed. The skewness ratio (skew/std error) and the kurtosis ratio (kurtosis/std error) were calculated using the range of −2 to +2 recommended by Pallant (2020). SEM was used to determine the relationship between neuroticism, conscientiousness, and academic motivation. We first tested the measurement models through confirmatory factor analysis, and second, we conducted a path analysis (in essence, the structural model: Schreiber et al., 2006). We inspected the goodness‐of‐fit statistics, which included the incremental fit indices (i.e., χ^2^, Tucker–Lewis Index [TLI], the Confirmatory Fit Index [CFI]) and the absolute fit indices (i.e., root mean square error of approximation [RMSEA] and standardized root mean square residual [SRMR]). Recommended cutoff values for overall fit were assessed (i.e., CFI and TLI ≤ 0.90, RMSEA and SRMR ≤ 0.08; Marsh et al., 2004). We applied the weighted least squares mean and variance adjusted (WLSMV) estimation method (Schumacker & Lomax, 2012). The WLSMV is a robust estimator that assumes nonnormality and is a good option for ordered data (Brown, 2006).

## 3 RESULTS

### 3.1 Preliminary analysis

Data screening preanalysis helped identify any missing data or participants who did not provide consent. To deal with missing data and determine if the items were MCAR, we implemented the expectation maximization strategy before analyzing the data. We determined the descriptive statistics and correlation coefficients for the Big‐5 factors and academic motivation and the correlations between academic motivation and the facets of neuroticism and conscientiousness. Additionally, we also determined whether the Big‐5 personality factors are related to academic motivation.

Pearson's product correlations were conducted by inputting the traits as independent variables and academic motivation as the dependent variable. The results from Table 1 reveal that conscientiousness had the strongest positive significant correlation with academic motivation (*r* = .60; 95% BCa CI [0.461, 0.714]; *p* < .001). Neuroticism had a significant weak correlation with academic motivation (*r* = −.28, 95% BCa CI [−0.442, −0.113]; *p* < .001). Surprisingly, openness to experience (*r* = .28, BCa CI [0.017, 0.435]; *p* = .02, *r* = .32, 95% BCa CI [0.117, 0.500]; *p* < .001) and extraversion (*r* = .26, 95% BCa CI [0.077, 0.428]; *p* < .01) all had a significant weak positive correlation with academic motivation.

**TABLE 1:** Descriptive statistics and correlation coefficients

	Mean	Standard deviation (SD)	1	2	3	4	5
Academic motivation	4.90	0.66	–	–	–	–	–
Openness	3.01	0.49	0.21**	–	–	–	–
Conscientiousness	3.19	0.57	0.44**	−0.31	–	–	–
Extraversion	3.43	0.60	22*	0.22**	0.20	–	–
Agreeableness	3.38	0.45	0.11*	0.22**	0.34*	0.04	–
Neuroticism	2.46	0.73	−0.19**	−0.08**	−0.53**	−0.39	−0.27

*Note*: *N *= 280. Bootstrapping results for all the variables were acceptable < 0.01.

Mean score averages were calculated to reflect the response rating scale.

* Sig. < .05.

** Sig. < .001.

#### 3.1.1 Hypothesis testing

Our hypothesized model, Model 1, included all the facets of conscientiousness and neuroticism, with academic motivation consisting of 13 latent constructs. Testing alternative models, Model 2 was a three‐factor model consisting of neuroticism, conscientiousness, and academic motivation. In contrast, Model 3 consisted of a two‐factor model of personality and academic motivation to perform.

Table 2 shows that Model 1, the 13‐factor model, fits the data best (χ^2^ (4016) = 30,970.68; CFI = 0.91; TLI = 0.91; RMSEA = 0.06; SRMR = 0.11[90% CI: 0.06, 0.07]) over the alternative three‐factor model (Model 2: χ^2^ (4019) = 34,379.26; CFI = 0.75; TLI = 0.74; RMSEA = 0.08; SRMR = 0.14 [90% CI: 0.08, 0.09]) and the two‐factor model (Model 3: χ^2^ (1014) = 34,279.25; CFI = 0.70; TLI = 0.70; RMSEA = 0.09; SRMR = 0.15; [90% CI: 0.09, 0.10]), supporting the objective of this study to consider the facets of conscientiousness and neuroticism.

**TABLE 2:** Fit statistic for the measurement model

	**χ^2^**	**Df**	**Tucker–Lewis Index**	**CFI**	**Root mean square error of approximation**	**Standardized root mean square residual**
Model 1: facet‐factor	30,970.68**	4016	0.91	0.91	0.06	0.11
Model 2: three‐factor	34,379.26**	4019	0.74	0.75	0.08	0.14
Model 3: two‐factor	34,279.25**	4186	0.70	0.70	0.09	0.15

** *p* < .005.

### 3.2 Facets of conscientiousness on academic motivation

Hypothesis 1 set out to determine if the conscientiousness facets of (a) self‐efficacy, (b) orderliness, (c) dutifulness, (d) achievement striving, (e), self‐discipline, and (f) deliberation have a positive direct effect on academic motivation.

Table 3 illustrates Pearson's product‐moment correlation coefficients between the facets of conscientiousness and academic motivation. The findings reveal that academic motivation was positively related to self‐efficacy (*r* = .48; *p* < .001), orderliness (*r* = .17; *p* < .0010), dutifulness (*r* = .24; *p* < .001), achievement striving (*r* = .48; *p* < .001), and self‐discipline (*r* = .33; *p* < .001), while deliberation had no significant correlation with academic performance.

**TABLE 3:** Descriptive statistics and correlation coefficients for the conscientiousness facets

	**Mean**	**Std**	**1**	**2**	**3**	**4**	**5**	**6**
Academic motivation	4.90	0.66	–	–	–	–	–	–
Self‐efficacy	3.80	0.75	.48**	–	–	–	–	–
Orderliness	3.10	0.99	.17**	.23	–	–	–	–
Dutifulness	3.81	0.61	.24**	.27**	.15*	–	–	–
Achievement striving	3.84	0.76	.48**	.51**	.30**	.37*	–	–
Self‐discipline	2.18	1.08	.33**	.50**	.40**	.27	.50**	–
Deliberation	2.40	1.10	.08	.18	.22**	.25**	.11*	0.18

*Note*: N = 98. Mean score averages were calculated to reflect the response rating scale. Bootstrapping results for all the variables were acceptable < 0.01.

* *p* < .05;

** *p *< .001.

Table 4 shows the path analysis (structural model) for the direct effects of the conscientiousness facets on academic performance. The results indicated that both self‐efficacy (*β* = 2.98, 95% BCa CI [1.80, 4.17]; *p* < .0001) and achievement striving (*β* = 2.78, 95% BCa CI [1.58, 4.17]; *p* < .0001) had a direct positive effect on academic motivation.

**TABLE 4:** Path analysis of conscientiousness facets on academic motivation

	**Estimate**	**Standard error (SE)**	**Est/SE**	***p***	**95% CI**
Intercept	119.80	11.45	10.46	.000	96.44, 141.53
Self‐efficacy	2.98	6.02	0.50	.000**	1.80, 4.17
Orderliness	4.02	4.13	0.97	.99	−0.81, 0.81
Dutifulness	5.77	6.61	0.87	.38	−0.72, 1.88
Achievement striving	2.78	6.11	0.45	.000**	1.58, 3.99
Self‐discipline	1.46	4.34	0.34	.74	−0.71, 1.00
Deliberation	−1.49	3.50	−0.43	0.67	−0.84, 0.54

*Note*: R^2^ = .306; *F*_(273)_ = 20.04. Hypothesis 1a and d is therefore accepted.

* *p* < .000.

### 3.3 Facets of neuroticism on academic motivation

Hypothesis 2 set out to determine if the neuroticism facets of (a) anxiety will have a positive direct effect on academic motivation, while (b) anger, (c) depression, (d) self‐consciousness, (e) impulsiveness, and (f) vulnerability will have a negative direct effect on academic motivation. Using Pearson's product‐moment correlations, neuroticism facets were put into a single block with academic motivation as the dependent variable.

The results in Table 5 showed that academic motivation had a significant weak negative correlation with self‐consciousness (*r* = −.19; *p* = .004), vulnerability (*r* = −.29; *p* < .001), and depression (*r* = −.21; *p* = .004), while anger, anxiety, and impulsiveness produced a nonsignificant result.

**TABLE 5:** Descriptive statistics and correlation coefficients for facets of neuroticism

	**Mean**	**Std**	**1**	**2**	**3**	**4**	**5**	**6**
Academic motivation	4.90	0.66	1					
Anxiety	3.29	1.10	−.04	1				
Anger	2.54	1.05	−.09	.58**	1			
Depression	2.64	1.16	−.21**	.65**	.46**	1		
Self‐consciousness	2.13	0.91	−.12**	.52**	.29**	.59**	1	
Impulsiveness	2.46	0.99	−.10	.27**	.24**	.23**	.15	1
Vulnerability	1.67	0.88	−.29**	.57**	.45**	53**	.50**	.21*

*Note. N* = 98. Bootstrapping results for all the variables were acceptable < 0.01. Mean score averages were calculated to reflect the response rating scale.

* *p* < .05;

** *p *< .001.

A path analysis was conducted to examine Hypothesis 2 further. Table 6 shows the path analysis (structural model) for the direct effects of the neuroticism facets on academic performance. The results indicated that anxiety (*β* = 1.99, 95% BCa CI [0.87, 3.10]; *p* < .0001) had a positive direct effect, while depression (*β* = −1.44, 95% BCa CI [−2.45, −0.43]; *p* < .0001) and vulnerability (*β* = −2.81, 95% BCa CI [−4.01, −1.62]; *p* < .0001) had a direct negative effect on academic motivation. The results also revealed that anxiety had a high partial and partial correlation with depression and vulnerability, indicating that the covariances anxiety, depression, and vulnerability likely resulted in the positive direct effect of anxiety on academic motivation. Hypothesis 2a,b, c is therefore accepted.

**TABLE 6:** Path analysis of neuroticism facets on academic motivation

	**Estimate**	**SE**	**Est/SE**	***p***	**95% CI**
Intercept	225.76	6.14	36.77	.000	213.67, 237.85
Anxiety^a^	1.99	0.57	3.49	.001**	0.87, 3.10
Anger	0.02	0.49	0.04	.97	−0.95, 0.99
Depression^b^	−1.44	0.51	−2.82	.005^*^	−2.45, −0.43
Self‐consciousness	0.29	0.53	0.55	.62	−0.86, 1.43
Impulsiveness	−0.47	0.43	−1.09	.27	−1.32, 0.37
Vulnerability^c^	−2.81	0.61	−4.61	.000**	−4.01, −1.62

^a^ Anxiety, partial *r* = .21; part *r* = .20.

^b^ Depression, partial *r* = −.17; part *r* = −.16.

^c^ Vulnerability, partial *r* = −.27; part *r* = −.26.

** *p* < .000; *p < .005 *R* ^2^ = .136; *F*_(273)_ = 7.167.

## 4 DISCUSSION

This study aimed to examine the correlation between conscientiousness, neuroticism, and academic motivation while also filling the gap in the available literature by exploring the facets. This paper's findings contribute to the literature by showing that conscientiousness has the strongest correlation with academic motivation, while neuroticism is negatively correlated. In line with the defensive pessimism argument, trait‐level anxiety (neuroticism facet) was the only significant positive predictor for academic motivation.

### 4.1 The conscientious facets are positively related to academic motivation

Hypothesis one stated that the facets of conscientiousness would all have a significant positive relationship with academic motivation. This study examined academic motivation, which Steinmayr et al. (2019) argued is a better predictor of grades than intelligence and previous grade scores. Consistent with previous literature examining academic performance (Chamorro‐Premuzic & Furnham, 2003; Hakimi Hejazi & Lavasani, 2011; Noftle & Robins, 2007; Trapmann et al., 2007), the results partially confirm that the facets of conscientiousness directly affect academic motivation. We found that the conscientious facets of achievement striving and self‐efficacy had a significant positive direct effect on academic motivation. This finding is consistent with Chamorro‐Premuzic and Furnham (2003) and Noftle and Robins (2007), who revealed that the conscientious facets of achievement striving and self‐efficacy produced the most considerable significant relationship with academic motivation. Considering that achievement striving characterises a hard‐working individual who aims to achieve their established goals, it is unsurprising that academic motivation is positively predicted. The explanation for the self‐efficacy trait may be found in Hayat et al. (2020), who argued that individuals high in self‐efficacy believed in their competence and consequently applied more effort to studying.

The only facet that did not significantly correlate with academic motivation was deliberation, which denotes long and careful consideration. Therefore, the results indicate that all the conscientious facets, except deliberation, are positively related to motivation.

### 4.2 The neuroticism facets are negatively related to academic motivation except for anxiety

Hypothesis 2 stated that all the facets of neuroticism, except anxiety, would negatively relate to academic motivation. Hypothesis 2 was partially confirmed, as the findings revealed that the neuroticism facets of vulnerability and depression were negatively related to academic motivation. The results complement the findings of Trapmann et al. (2007), and O'Súilleabháin et al. (2019) argued that the vulnerability trait negatively correlated with adapting to recurring stress and resulted in the sense of hopelessness. Perhaps the vulnerability facet negatively predicts academic motivation due to the individual feeling unable to manage the stress of studying, and the sense of hopelessness might negatively impact their motivation.

The depression facet may be related to difficulty finding motivation when experiencing a low mood. Individuals scoring high in depression are also characterized as having a low opinion of themselves, as evidenced in Costa and McCrae (1992), which may explain why they are not motivated to study, as they may not believe that they can excel academically. Symptoms of depression also include low mood, apathy, discontent, loss of interest, inactivity, and lack of concentration (American Psychiatric Association, 2013), contributing to the lack of motivation in general to perform. Amsterdam et al. (1987) further note that people with neurotic depression are likely emotionally unstable. Therefore, it is expected that performance‐related activity might produce emotional responses that, if not managed, could lead to reduced motivation in activities (e.g., academic motivation).

We hypothesized that the defensive pessimism produced by the anxiety facet would result in this facet being positively related to academic motivation. Anxiety was the only significant positive predictor for academic motivation when controlling for the other neuroticism facets using SEM. The anxiety facet produced a significant positive result because it relegated and interacted with other facets. The only other significant facets were vulnerability and depression, which were both demonstrated to be negative predictors of academic motivation, which partially falls in line with previous results from Trapmann et al. (2007).

Khalin (2020) investigated internalizing disorders and demonstrated that anxiety and depression had the highest comorbidity rate. Furthermore, anxiety disorders appear to precede the development of depression disorders (Khalin, 2020), suggesting that if anxiety is not managed, it could cause the onset of depression. Therefore, the SEM results show that anxiety is positively related to academic motivation when controlling for the other facets. In contrast, depression is negatively correlated, although there is a covariance between them. As argued earlier, the finding that anxiety predicts academic motivation may indicate a motivating factor in anxiety‐induced defensive pessimism.

Defensive pessimism due to anxiety is defined as fearing for the worst (Norem & Chang, 2002), leading to compulsive preparation (Komarraju & Karau, 2005). Although this paper investigated motivation rather than performance, this finding supports the argument put forward by Trapmann et al. (2007), which stated that the neuroticism facets generate different contributions to academic performance. The different contributions are encapsulated by the vulnerability and depression facets producing a negative relationship, while anxiety produced a positive relationship with academic motivation. Therefore, Pearson's correlation and SEM results address the literature gaps by demonstrating that the facets of neuroticism provide different correlational and predictive contributions.

### 4.3 Practical implications

This study was conducted to determine how induvial personality differences could motivate individuals to study. Understanding motivation is critical due to the university landscape changing to online in response to the COVID‐19 pandemic. The findings in this paper indicate that conscientious individuals are the most motivated to study. Scoring high in neuroticism appears to negatively correlate with motivation, decreasing students' motivation to study.

Examining the facets of neuroticism using SEM demonstrated that only the anxiety facet positively predicts motivation. One potential practical takeaway is that, regardless of their level of conscientiousness, if a student is anxious, they might be motivated to study because of their fear of failing the unit. Although anxiety can predict academic motivation irrespective of conscientious levels, the vulnerability and depression facets have the opposite relationship. Therefore, anxiety serves as a motivator to study, but if left unchecked, it could lead to depression, which is detrimental to motivation.

### 4.4 Limitations and future research

This paper focuses on how the Big‐5 traits and facets relate to and predict academic motivation; therefore, our findings are limited to the academic setting. Future research should examine the Big‐5 and motivation in different domains like sports or occupation. Although this paper supported the findings from previous literature and presented an interesting finding on anxiety, future research should conduct more analyses using a larger sample size. Future research should examine the relationship between the other traits, such as perfectionism. For example, Stoeber et al. (2009) demonstrated that conscientiousness predicts perfectionism. Therefore, future research should investigate the relationship between traits and their facets to clarify their contributions to academic motivation and performance.

An additional limitation is the gender skew in this present paper, which had 78.2% of this study's participants identify as female. This gender skew presents issues with generalizability. This skew may be due to the Bachelor of Psychology Course; therefore, future research should investigate how the Big‐5 traits and facets affect motivation in other college courses and careers.

This paper was also conducted during COVID‐19, which may have inflated the neuroticism results. Future research may need to explore the relationship between COVID‐19 and the facets of neuroticism like anxiety and depression, and how they affect motivation.

## 5 CONCLUSION

In summation, the conscientiousness facets of achievement striving and self‐efficacy strongly correlated with academic motivation. Neuroticism and most of its facets were negatively correlated with motivation. However, the SEM path analysis demonstrated that anxiety is a significant positive predictor of academic motivation when controlling for the other facets. Therefore, in line with our defensive pessimism argument, a one‐point increase in anxiety leads to a 1.99 increase in academic motivation. Furthermore, this result supports the idea that irrespective of conscientious levels, anxiety leads to motivation due to defensive pessimism. Previous research has argued that anxiety may lead to overpreparation due to imagining the worst‐case future scenario. For example, if a student imagines that they will fail an exam or assessment, this fear of the worst may motivate them to study. This type of defensive pessimism provides one potential explanation for why only the anxiety facet of neuroticism positively predicted academic motivation.

On the other hand, the vulnerability and depression facet of neuroticism produced a significant negative result in the correlational and SEM analysis. Complementing previous research, this paper revealed that vulnerability negatively predicts and correlates with academic performance due to individuals being unable to manage stress adequately. This paper expands on previous literature by examining the facets of neuroticism and academic motivation rather than GPA scores using an Australian sample studying online due to COVID‐19 and implementing an SEM analysis. Therefore, although there is a covariance between the neuroticism facets of vulnerability, depression and anxiety, their contributions to motivation are unique and deserve further attention.

## CONFLICTS OF INTEREST

The authors declare that there is no conflict of interest.

### PEER REVIEW

The peer review history for this article is available at: https://publons.com/publon/10.1002/brb3.2673.

## NOTE

1 The data that support the findings of this study are openly available in [google drive] https://drive.google.com/drive/folders/1040ABU0Tf6Flr75K6uMIVC7Z3q2d0zmn?usp=sharing reference number HREC 711041220
